# The Variability of Refractivity in the Atmospheric Boundary Layer of a Tropical Island Volcano Measured by Ground-Based Interferometric Radar

**DOI:** 10.1007/s10546-016-0168-3

**Published:** 2016-06-22

**Authors:** G. Wadge, A. Costa, K. Pascal, C. Werner, T. Webb

**Affiliations:** 1 Department of Meteorology, University of Reading, Reading, RG66AL UK; 2 Instituto Nazionale di Geofisica e Vulcanologia, 40128 Bologna, Italy; 3 Montserrat Volcano Observatory, Flemmings, Montserrat; 4 Gamma Remote Sensing Research and Consulting AG, 3073 Gümligen, Switzerland

**Keywords:** Ground-based radar interferometer, Island boundary layer, Refractivity, Water vapour

## Abstract

For 24 h we measured continuously the variability of atmospheric refractivity over a volcano on the tropical island of Montserrat using a ground-based radar interferometer. We observed variations in phase that we interpret as due to changing water vapour on the propagation path between the radar and the volcano and we present them here in the context of the behaviour of the atmospheric boundary layer over the island. The water vapour behaviour was forced by diurnal processes, the passage of a synoptic-scale system and the presence of a plume of volcanic gas. The interferometer collected images of amplitude and phase every minute. From pairs of phase images, interferograms were calculated and analyzed every minute and averaged hourly, together with contemporaneous measurements of zenith delays estimated from a network of 14 GPS receivers. The standard deviation of phase at two sites on the volcano surface spanned a range of about 1–5 radians, the lowest values occurring at night on the lower slopes and the highest values during the day on the upper slopes. This was also reflected in spatial patterns of variability. Two-dimensional profiles of radar-measured delays were modelled using an atmosphere with water vapour content decreasing upwards and water vapour variability increasing upwards. Estimates of the effect of changing water vapour flux from the volcanic plume indicate that it should contribute only a few percent to this atmospheric variability. A diurnal cycle within the lower boundary layer producing a turbulence-dominated mixed layer during the day and stable layers at night is consistent with the observed refractivity.

## Introduction

Steep terrain and high humidity are common on volcanic islands in the tropics. They tend to create strong, variable gradients of water vapour that in turn lead to local variations in the refractive index of the air and complex three-dimensional patterns of refractivity. The refractivity of the troposphere is controlled mainly by the temperature, the partial pressure of dry air, the partial water vapour pressure, and the liquid water content (Smith and Weintraub 1953; Thayer 1974). Water vapour is the main source of the variability of refractivity within the atmospheric boundary layer (ABL) (Stull 1988) over length scales from metres to a few kilometres and over intervals as short as a minute. This variability affects the measurement of microwave signals that pass through such fields, as in the case of interferometric synthetic aperture radar (InSAR; Hanssen 2001), which uses differential phase measurements to detect land-surface motion.

**Fig. 1:**
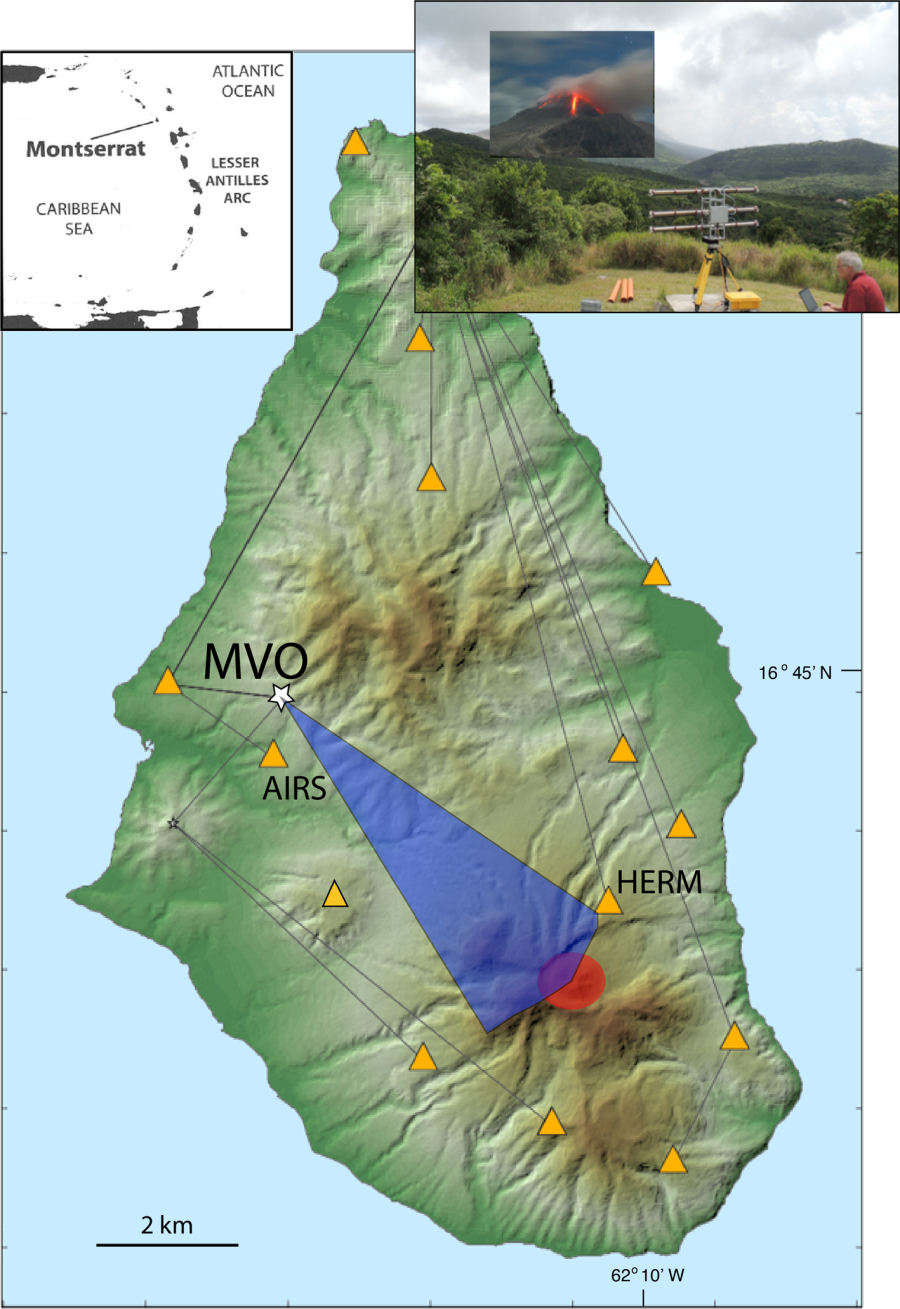
Location of the GPRI measurement site (*white star*, labelled Montserrat Volcanic Observatory) on a *shaded* relief map of Montserrat (*inset* map shows location in Lesser Antilles). The *inset* photograph is of the GPRI in operation at the Montserrat Volcanic Observatory. *Yellow triangles* are the GPS receiver sites. The Soufrière Hills Volcano lava dome is shown by the *red area*. The *blue area* shows the GPRI field of sight from the Montserrat Volcanic Observatory. The AIRS and HERM GPS receiver sites lie just outside this area and sample part of the GPRI measurement space

In 2012 and 2013 we undertook a series of measurements using a ground-based interferometric radar named the Gamma Portable Radar Interferometer (GPRI-2, Werner et al. 2012, GPRI hereafter) on the Soufrière Hills Volcano of Montserrat in the Lesser Antilles island arc of the eastern Caribbean (Fig. 1). The radar phase signal generated by GPRI, when multiplied by the complex conjugate, geometrically equivalent, signal from some later time, provides a mapping of phase difference, via instrument scanning, along the lines-of-sight from the instrument to the ground at a distance. In our case these radar propagation paths vary from horizontal to $$+8^{\circ }$$. The phase difference (or delay relative to the velocity through a vacuum) along the slant path of radar propagation through the atmosphere, $$S_k^{t_i } $$ , at acquisition time, $$t_{i}$$, for resolution element, *k*, is given by the integrated refractivity from the radar to the ground,

1 $$\begin{aligned} S_k^{t_i } =10^{-6}\int _0^H {\frac{N}{\cos \theta _\mathrm{inc} }\mathrm{d}h} \end{aligned}$$

where *N* is the refractivity, $$\theta _\mathrm{inc} $$ is the incidence angle measured from the zenith, and $$h_0^H $$ is the height of the radar above the ground at *k* (Hanssen 2001). Two such acquisitions are required to form an interferogram. We ignore the effect of any changes in path curvature over these distances (Bean and Dutton 1968), and the ratio of the radar phase differential delay (mm) to the water vapour content (mm) is often taken as a constant (Bevis et al. 1992), here as 7.0.

The use of satellite-based radar interferometry to monitor the deformation of the surface of volcanoes due to magma transport is used increasingly by volcano observatories. But water vapour variability in the ABL is recognised as the major source of uncertainty in these measurements (e.g. Lu and Dzurisin 2014). Improved understanding of changes in refractivity due to water vapour would be of great benefit in this regard.

The purpose of our study is to test the idea that these ground-based radar measurements of changes in water vapour can be related to the nature and behaviour of the ABL over a steep-sided volcano in the humid tropics. In other studies of the effects of the troposphere on the signal from ground-based interferometers, the path lengths have been much shorter (typically $\approx \,1$ km) and through the mid-latitude atmosphere (e.g. Iannini and Monti Guarnieri 2011). To our knowledge this is the first study of the delay effects on ground-based radar over longer paths ($\approx$5 km) through a humid tropical atmosphere. Our study was conducted from a single site over a period of 24 h, from 1020 local time (LT) on 4 August to 1020 LT on 5 August 2013. Based on the lack of volcanic activity and the known background rates of surface deformation on Montserrat (Odbert et al. 2014) we assume that over this short period we can ignore any volcano-related surface motions.

We anticipate three types of forcing that should affect the water vapour in the ABL in our case: a diurnal cycle of radiant heating and cooling of the island that drives orographically-modulated processes, the effects of the plume of hot gas from the volcano, and a synoptic-scale change in the atmosphere. In Sect. 2 we present a brief overview of the expected characteristics of water vapour within the boundary layer over a tropical island. In Sect. 3 we describe the radar and the data that were collected and in Sect. 4 we present the interferometric data collected continuously over the 24-h period. These are then analyzed in Sect. 5 in terms of different length and time scales of observation, and a summary is presented in Sect. 6 focusing on whether the results fit a conceptual model of the ABL.

## The Boundary Layer and Water Vapour

Atmospheric refractivity at radar wavelengths can be modulated locally by spatial changes in pressure, temperature, water vapour and by hydrometeors. The effect of pressure variations on refractivity is gradual and of low variability over scales of $\sim 10\hbox { km}$, as in our case, and can be ignored. The relative sensitivity of refractivity to temperature and water vapour over ranges of temperature ($+40$ to $-40\,^{\circ }\hbox {C}$) and partial pressure of water vapour (0 to 30 hPa) was studied by Hanssen (2001, Fig. 6.2). The sensitivity ratio of water vapour change (1 hPa) to temperature change ($1\,^{\circ }\hbox {C}$) is between 4- and 20-fold. The two variables act in opposite senses, an increase in water vapour pressure by 1 hPa increases refractivity by 4–20 times the reduced refractivity due to an increase of $1\,^{\circ }\hbox {C}$ in temperature. Water vapour pressure variations of 1 hPA are common over scales of 1 km and tens of minutes. Liquid water causes a much smaller refractive delay than an equivalent mass of water vapour and can be ignored except in conditions of very high rainfall rates (Hanssen 2001, Table 6.1), which we do for 4–5 August 2013. Thus for our analysis we focus on water vapour effects. The vertical content of water vapour generally decreases exponentially (Foster and Bevis 2003), with over 99 % of water vapour content occurring in the lowest 10 km of the troposphere, mostly within the ABL.

**Fig. 2:**
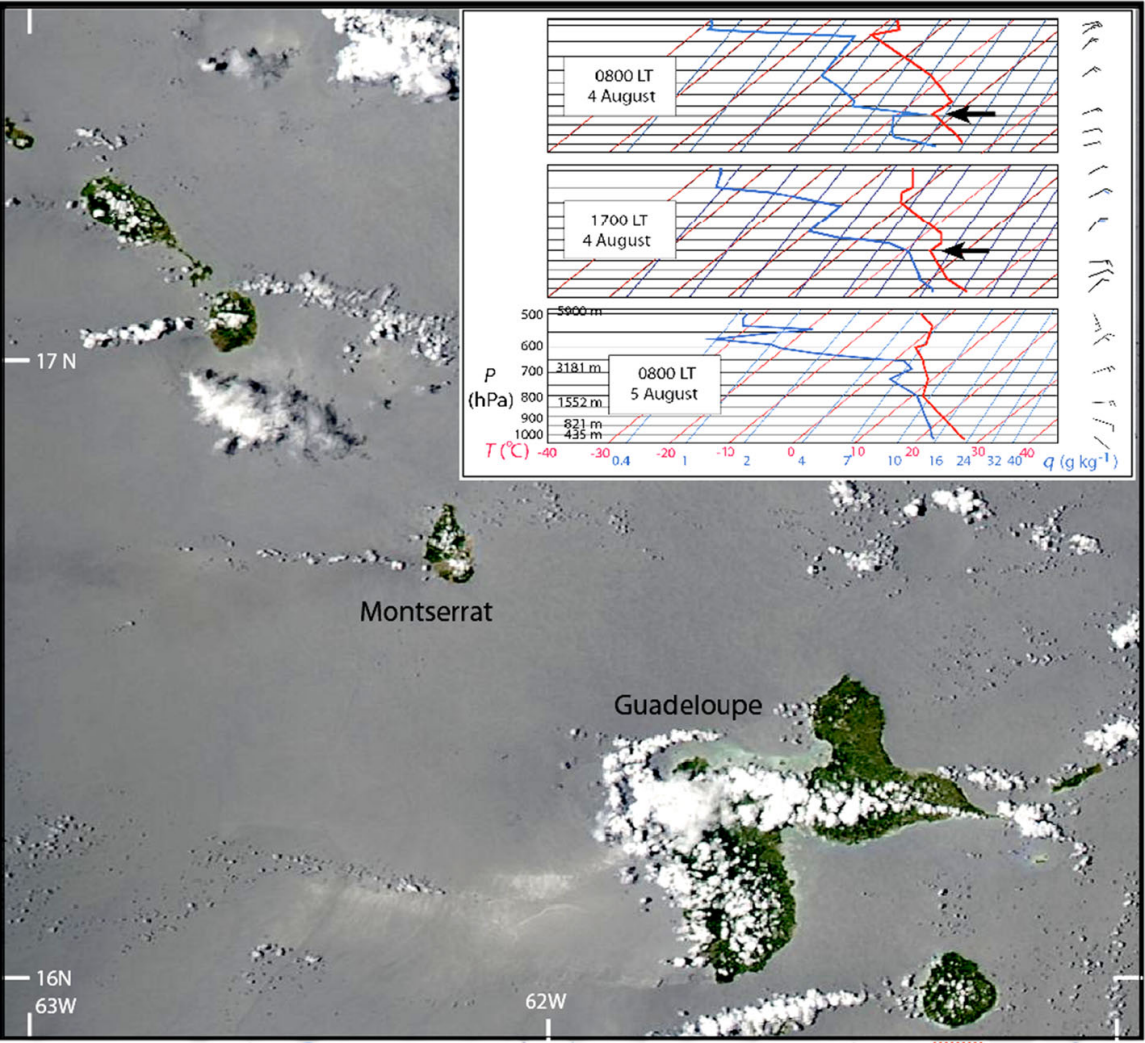
MODIS Aqua image of Montserrat at 1330 LT 5 August 2013. Note the east-west cloud trails, but no lee waves. *Inset* are three skew $T (x\hbox {-axis},\, ^{\circ }\hbox {C}) - \hbox {log}P$ (*y*-axis, hPa) plots of radiosonde data from Le Raizet, Guadeloupe at 0800 and1700 LT 4 August and 0800 LT 5 August. The specific humidity (*q*) values are shown in *blue*. *Arrows* show the positions of temperature inversions

The climate of Montserrat is dominated by the surrounding ocean and the easterly trade wind circulation. The ABL over the sea to the east (upwind) of the Lesser Antilles was observed intensively in the Barbados Oceanographic Meteorological Experiment (BOMEX) campaign of 1974 and later simulated (Siebesma et al. 2003). During the day it consists of a basal surface layer dominated by evaporation, an uppermost entrainment layer where cool air from the free troposphere descends and a deep, mixed layer between, where turbulence-driven cumulus clouds are generated. More recent work resulting from the Rain in Cumulus over the Ocean (RICO) project, to the north-east of Montserrat, has emphasised a more varied conceptual picture than that of BOMEX (Davison et al. 2013). In the winter months, the ABL can extend up to 4 km in altitude with highly variable relative humidity values and weaker development of temperature inversions.

The ABL inherited from the tropical Atlantic Ocean becomes more complex over the islands of the Lesser Antilles. Moist air forced to rise over the mountains produces clouds and sometimes rain, and the timing and location of precipitation has been studied in detail on the island of Dominica, 200 km south of Montserrat, and depends generally on the low-level wind speed, mechanically-triggered convection and the laterally heterogeneous fields of moisture-rich volumes of the windward airflow (Smith et al. 2009, 2012; Kirshbaum and Smith 2009; Minder et al. 2013). The lifting condensation level on Dominica and Montserrat typically varies around 600 m above sea level (a.s.l.) and the top of the entrainment zone is about 2000 m a.s.l.

The structure of the ABL may vary considerably during the diurnal cycle, particularly over land in the tropics (Stull 1988), with abrupt changes to the turbulent regime starting at sunset and sunrise. The top of the ABL can often be detected in radiosonde data by the presence of a temperature inversion, and Fig. 2 shows such inversions at 1600 and 1850 m a.s.l. detected by the radiosondes released on 4 August 2013 from the island of Guadeloupe, 100 km south-east of Montserrat. Topography also plays a major role in the behaviour of the boundary layer. Soufrière Hills Volcano has a steep and rugged lava dome at its summit (from 800 to 1080 m a.s.l.) with slopes of talus derived from the dome and radial valleys incised into the lower slopes. Sea breezes and valley winds in the morning and their nocturnal equivalents may modify the boundary layer during the diurnal cycle, but have not been studied at Montserrat. Adler and Kalthoff (2014) showed, from measurements of the vertical structure of the specific humidity field in the mountains of Corsica, that the sea-breeze front effectively lifted the upper surface of the ABL by about 1.5 km over a period of about 6 h. On Dominica, a dry plunging flow to the west with a pinched ABL and humidity field can be formed just leeward of the mountains during favourable flow conditions (Minder et al. 2013). Katabatic flow downhill to the windward (east) is known from Guadeloupe (Cécé et al. 2014). Montserrat, like many of the volcanic islands of the Lesser Antilles, is susceptible to cloud trails forming by convergence in the wake of the heated islands (Kirshbaum and Fairman 2015) (Fig. 2).

We might expect to find different spatial distributions of water vapour at night from those during the day. During the day, as a result of strong convective turbulence in the mixed layer, we would expect to see more rapid fluctuations in path delays, particularly at higher elevations. During the night the GPRI propagation paths should have passed through part of the stable layer, which has much reduced levels of turbulence and hence should have much lower variability of refractive path delays. The transitions between the day and night states are times of rapid structural evolution in the ABL, particularly the morning transition, which can last for 2–3 h (e.g. Ketzler 2014).

## Interferometric Data

### Montserrat

**Fig. 3:**
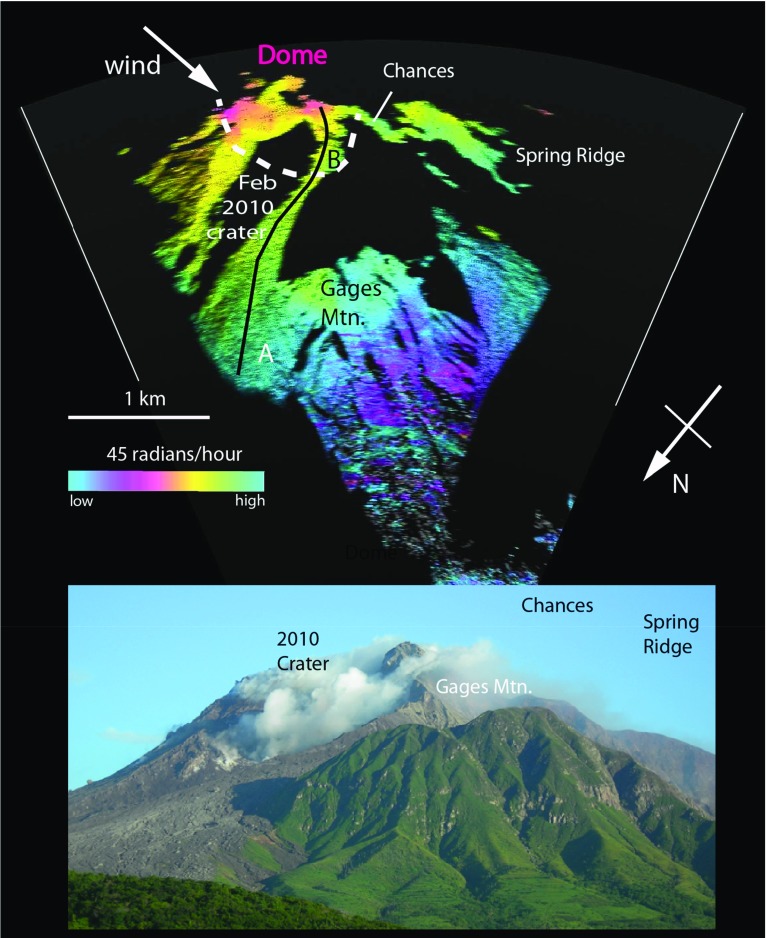
An interferogram taken from Montserrat Volcano Observatory (on 5 October 2012) towards Soufrière Hills Volcano, shown as a polar plot subtending an angle of $45^{\circ }$ (*solid white lines*). Note the *black areas* are those occluded from radar returns. The phase differences recorded over an hour is shown by the colours (see scale) modulated by a *grey scale* representing the amplitude of radar backscatter from the ground surface. The *dashed white line* indicates the rough position of the lava dome. A *white arrow* indicates the direction of the trade winds over the period of interest. *A* and *B* are measurement sites on the talus slope and the *black line* is the profile used in Fig. 7. Below is a photograph taken from the same position at a time of no meteorological cloud. The cloud visible is the volcanic plume rising from vents in the crater formed on 11 February 2010 and being carried westward, to the right, by the trade winds

We deployed the GPRI on Montserrat during two periods: 1–5 October 2012 and 1–13 August 2013, though here we mainly use data from 4–5 August 2013. Montserrat ($17^{\,\circ }\hbox {N}$, $62^{\,\circ }\hbox {W}$) is a small (16 km $\times$ 10 km) tropical island with a wet season (July–November) and a typical surface temperature range from 24 to $33\,^{\circ }\hbox {C}$. Measurements on 4–5 August using the GPRI were made from the Montserrat Volcano Observatory site (MVO; elevation 279 m a.s.l.) towards Soufrière Hills Volcano, which dominates the southern part of the island and produced a long, complex eruption between 1995 and 2010 (Wadge et al. 2014) (Fig. 1). The volcano has a summit elevation of about 1080 m a.s.l. and a basal diameter of about 10 km at sea level. The uppermost flanks of the volcano have slopes of $20^{\circ }$–$40^{\circ }$, with local cliffs and are largely rocky or covered in ash. Below them are talus slopes, whilst the lower flanks have slopes often less than $10^{\circ }$ and are covered by vegetation in places. We chose two measurement sites on the vegetation-free talus slopes (A and B, Fig. 3) from which we extracted statistics of the radar returns. These sites were too hazardous to permit deployment of instrumental reflectors. The character of these sites comprises ash surfaces with several decimetre- to metre-sized blocks of lava in each resolution element (30–40 m across) that act as stable reflectors. This was evident in the stability of the amplitude values over the day. Also the correlation of the phase variability in time with the diurnal cycle showed that this was largely driven by the atmosphere not surface noise.

Although no new lava has been produced since February 2010, a plume of gases is emitted continuously at variable rates. Most of this gas is water vapour, derived partly from magma via ex-solution at depth and from hydrothermally-mobilised groundwater. This water-rich plume is usually advected to the west by the trade winds, diffusing and depositing mass (Rodriguez et al. 2008), as was the case during these measurements.

### GPRI and Ancillary Measurements

The GPRI is a real-aperture terrestrial radar that was mounted on a tripod whose feet could be screwed to a base for stability and accurate re-occupation (Fig. 1, inset). The radar operates in the range 17.1–17.3 GHz (Ku-band) using a frequency modulated continuous wave chirp. There are separate 2.1 m long, horizontally mounted antennae, one that transmits and two that receive (though only one was used here), each with a $35^{\circ }$ beam width in elevation. A motorized gimbal moves the antenna assembly about a vertical axis to scan the terrain at a rate of about $10^{\circ }\hbox { s}^{-1}$, giving a range resolution of 0.95 m and an azimuthal resolution of $6.8\hbox { m km}^{-1}$ . When not scanning, the azimuthal resolution of the radar is reduced to $8.75\hbox { m km}^{-1}$, the operational range interval is from 50 m to about 10 km, and a co-axial GPS receiver provides timing and location signals. In practice, we acquired most of our images at intervals of about 1-min over ranges of 2–6 km and within an azimuthal arc of $45^{\circ }$ (Fig. 3). Strong winds can create vibrational noise without a radome (our case). In the 2013 campaign we used a Kestrel 4500 portable weather station to record site temperature, pressure and relative humidity, and also made phenomenological observations about the state of the atmosphere (e.g. rain showers). Wind speed was recorded in daylight hours from the airport, 10 km north of the volcano. Radiosonde measurements from Le Raizet, Guadeloupe, were also available twice a day (Fig. 2). The Montserrat Volcano Observatory maintains a network of 14 GPS receivers across the island (Fig. 1) and routinely calculates the zenith wet delay (*ZWD*) values due to water vapour, using GAMIT-GLOBK software to process the raw GPS data . Here we use *ZWD* data computed at 5-min intervals, using globally-averaged values for temperature and pressure.

### Nature of the GPRI Interferograms

If the atmosphere traversed by the GPRI’s beam during two image acquisitions remains completely stable then the resulting interferogram displays a zero phase difference. Any change of water vapour content along the radar path from one image to another is recorded as an integrated phase difference. Changes in water vapour distribution are usually gradual, producing a spatially correlated pattern of phase gradients. Two geometric factors may affect the resulting phase difference image. Firstly, the longer the path to the ground surface from the radar, the greater the cumulative path and the greater the likelihood of encountering phase fluctuations along that path. Secondly, because the diameter of the radar’s footprint increases with range (by $7\hbox { m km}^{-1}$), a water vapour fluctuation of a given shape in the near range affects more of the image than the same fluctuation at far range. In addition, Iannini and Monti Guarnieri (2011, Fig. 2) showed that water vapour variability generally increases with increasing wind speed.

The wavelength of the GPRI radar is 17.43 mm and thus a full phase cycle equates to 1.386 mm of delay per radian of phase change. Also one radian equates to approximately 0.2 mm of precipitable water vapour (PWV) along the radar path. Figure 3 shows an interferogram of the volcano generated by averaging the rate of change of phase difference over 1-h (60 scans) from the Montserrat Volcanic Observatory site (Fig. 1). The statistical details of these changes are presented in the next section for the 4–5 August 2013 data. The near-range lower slopes are forested and are partly incoherent. There are also a number of black areas where the radar signal is occluded, particularly behind Gages Mountain and at the collapse crater formed by the last eruption of the volcano on 11 February 2010. The skyline of the image comprises Spring Ridge, Chances and the lava dome, while the approximate location of the visible part of the lava dome is shown by the white dashed line in Fig. 3. The gradient of phase change seen in this interferogram is typical of the data more generally, varying with range distance from the radar to the surface.

**Table 1:** Observed conditions from 1020 LT on 4 August to 1020 LT on 5 August 2013

Date	Site	Elevation (m a.s.l.)	Time (LT)	Local conditions	Regional conditions
4 August	MVO	280	1020	600 m a.s.l. cloud base	Topographic cloud, lee waves
			1040	Brief rain shower	
			10–1800	Wind speeds fall from 9 to $5\hbox { m s}^{-1}$	
5 August			0300	Rain shower, humidity rising	No lee waves. Arrival of moist air from south
			0600	700 m a.s.l. cloud base	
			0647	Small volcanic earthquake	
			0800	Rain shower	

## Measurements on 4–5 August 2013

The synoptic weather conditions changed during the observation period (Table 1). The morning of 4 August marked the end of a period of stable conditions with relatively strong easterly trade winds that produced distinct lee wave clouds to the west of the mountainous islands in the northern Lesser Antilles, including Montserrat on 2 and 3 August. The surface wind speeds on Montserrat decreased from about $9\hbox { m s}^{-1}$ early on the morning of 4 August, to $5\hbox { m s}^{-1}$ by the evening (Fig. 8) and the lee wave clouds disappeared from MODIS satellite images. GOES-13 water vapour imagery showed that a west-south-west to east-north-east oriented ridge of low water vapour moved northwards during this 24-h period, bringing more humid air over Montserrat behind it.

### Interferometric Radar

Between 1020 LT (UTC—4 h) on 4 August and 1020 LT on the 5 August 2013, we acquired images of radar amplitude and phase every minute from the Montserrat Volcano Observatory site, about 5 km north-west of the volcano (Fig. 1). One-minute interferograms were processed from consecutive phase image pairs (1–2, 2–3...). To provide an accessible summary, these 1-min interferograms were averaged in blocks of 1-h stacks, starting and finishing at 20 min past the hour. The colour cycle used for these interferograms is blue to purple to magenta to orange to yellow to green with increasing phase difference (Fig. 3). Those images with hues changing rapidly over short distances record significant net changes in the water vapour of the atmosphere at the kilometre and sub-kilometre scale over that hour, or are subject to poor phase unwrapping. Phase unwrapping is the process in which the ambiguities of the phase field across $2\uppi$ boundaries are identified and a continuous field created across them. Errors during this unwrapping process may occur where the data are too incoherent or the phase field is locally too complex relative to the resolution cell. The phase rate standard deviation (p.r.s.d.) of the interferograms gives a measure of the temporal variability of the phase differences.

### GPS

**Fig. 4:**
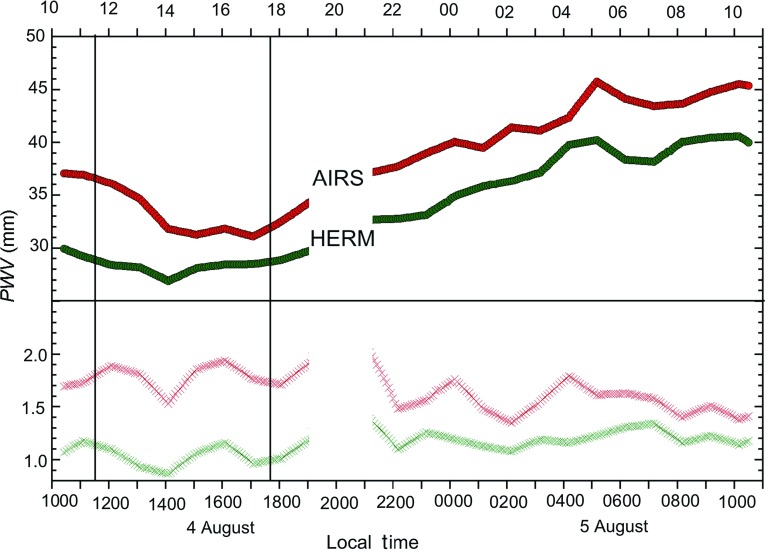
*Top panel* time series of *PWV* data recorded by the AIRS and HERM GPS receivers for the period 1020 LT 4 August to 1020 LT 5 August 2013. *Bottom panel* the equivalent standard deviations of the *PWV* values. The gap between 1900 and 2100 LT represents inaccurate data due to nature of the 24-h GPS processing cycle. The two *vertical lines* are the times framing the interferogram shown in Fig. 5

Two of the Montserrat Volcano Observatory GPS receivers (AIRS and HERM) lie close to the paths observed by the GPRI radar (Fig. 1). The AIRS receiver sits about 1 km south of the Montserrat Volcano Observatory at an elevation of 119 m a.s.l., within the Belham River valley, but is occluded from the radar. The HERM receiver lies north-east of the dome (Fig. 1), at an elevation of 472 m a.s.l., near the lower eastern skyline of the image shown in Fig. 3. It is the second highest GPS site on the volcano and is closest in location and elevation to point A (Fig. 3). The measurement space from the GPS receivers to the GPS satellites is an inverted cone with an elevation angle cut-off at $10^{\circ }$ from the horizontal. Both of these receivers sample part of the atmospheric volumes intersected by the radar in the very near range for the AIRS receiver and in the mid-range for the HERM receiver. In Fig. 4, the time series of *PWV* data, derived from *ZWD* values, for both AIRS and HERM receivers are shown. These values represent the water vapour contents integrated vertically through the whole atmosphere, in contrast to the sub-horizontal sampling through the ABL given by the ground-based radar.

Over the 24-h period, the two GPS receivers recorded *PWV* values that increased by 13–14 mm (91–98 mm of delay) (Fig. 4). The *PWV* curves of the two instruments are similar, falling to minima during the afternoon of 4 August and then rising in a fluctuating manner for the next 12 h. *PWV* values are about 5 mm lower for the HERM site relative to the AIRS site, reflecting the 350 m greater elevation of the HERM site. Both the AIRS and HERM sites show maxima in *PWV* at about 0500 LT on 5 August (Fig. 4), and the AIRS record shows inflections from 0000 LT onwards and HERM from about 0300 LT, suggesting the transit of short wavelength water vapour features across the island. The standard deviation of the *PWV* measurements shown in the lower panel of Fig. 4 varies from 1 to 2 mm (7 to 14 mm of delay) and is almost twice as large for the AIRS site as for the HERM site for most of 4 August, but the contrast lessens as more humid air arrives later.

### Other Measurements

At Guadeloupe, three radiosondes were launched during our measurement period (Fig. 2). At 0800 LT on 4 August a clear temperature inversion zone at the top of the ABL was recorded at an altitude of about 1600 m a.s.l. By 1700 LT on 4 August this inversion feature had risen to about 1850 m a.s.l. These are values we would expect to see based on the measurements and models of Siebesma et al. (2003). However, the next radiosonde at 0800 LT on 5 August shows no evidence of a true inversion. The measured ABL winds shifted from east-north-east to east to east-south-east during the 24-h period. On Montserrat, recorded wind speeds decreased from about $9\hbox { m s}^{-1}$ to about $5\hbox { m s}^{-1}$ between 1000 and 1800 LT on 4 August. Temperature, pressure and relative humidity values were recorded at the Montserrat Volcano Observatory site. Relative humidity rose sharply from about 70 % during the daytime on 4 August to 80–85 % during the night, with the pressure stable around 980 hPa. There was a gradual cooling during the hours of darkness from 1800 LT to about 0600 LT, as we might expect. But this was interrupted at about 0300 LT, and about 0800 LT by sinusoidal perturbations with amplitudes of up to 1–2 $^{\circ }\hbox {C}$ (Fig. 8).

## Analysis of the Radar Data

In the following four sub-sections we address the questions: what is the structure of the refractivity field over the volcano? How does it vary from minute to hour to daily scales? What role does the volcanic plume play in refractivity change?

### Multi-hour Interferogram and a Stratified Refractivity Model

**Fig. 5:**
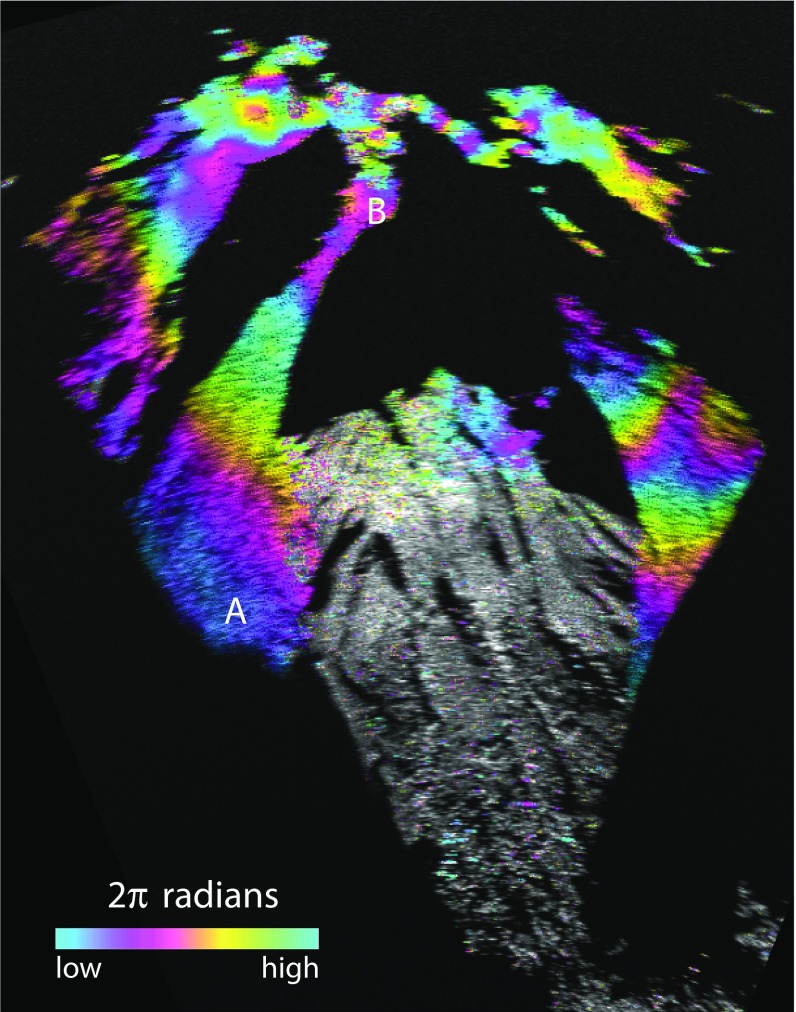
A 6-h, (1130–1740 LT) 4 August 2013, interferogram. Phase colour is merged with intensity information (*grey scale*). *A* and *B* are sites on the profiles in Figs. 8 and 9. One cycle of colour represents $2\uppi$ radians of phase change

Here we analyze a single interferogram constructed from two phase field images 6 h apart (Fig. 5). The high coherence of much of the non-vegetated surface of the volcano is a result of the stability of the surface and the strong spatial correlation of the water vapour field over distances of tens to hundreds of metres. The choice of these two particular images has no significance except that the resulting interferogram is typical of non-averaged data, based on visual interrogation. We assume that all of the phase delay is produced in the atmosphere and compare it with a simple model of refractivity that decreases linearly upwards but is uniform horizontally (Iglesias et al. 2014).

Figure 5 shows the interferogram constructed from images collected at 1130 LT and 1740 LT on 4 August. The vegetated Gages Mountain (Fig. 3) and foreground slopes phase difference signals are decorrelated, but the signals of the talus surfaces to the east and west are coherent. There is a gradient of phase that increases by about 8 radians (11 mm) up the slope to the east of Gages Mountain from site A to B, about 400 to 800 m a.s.l. respectively. Above that, the phase flattens off and then decreases steeply over the lava dome where the signal is noisier.

**Fig. 6:**
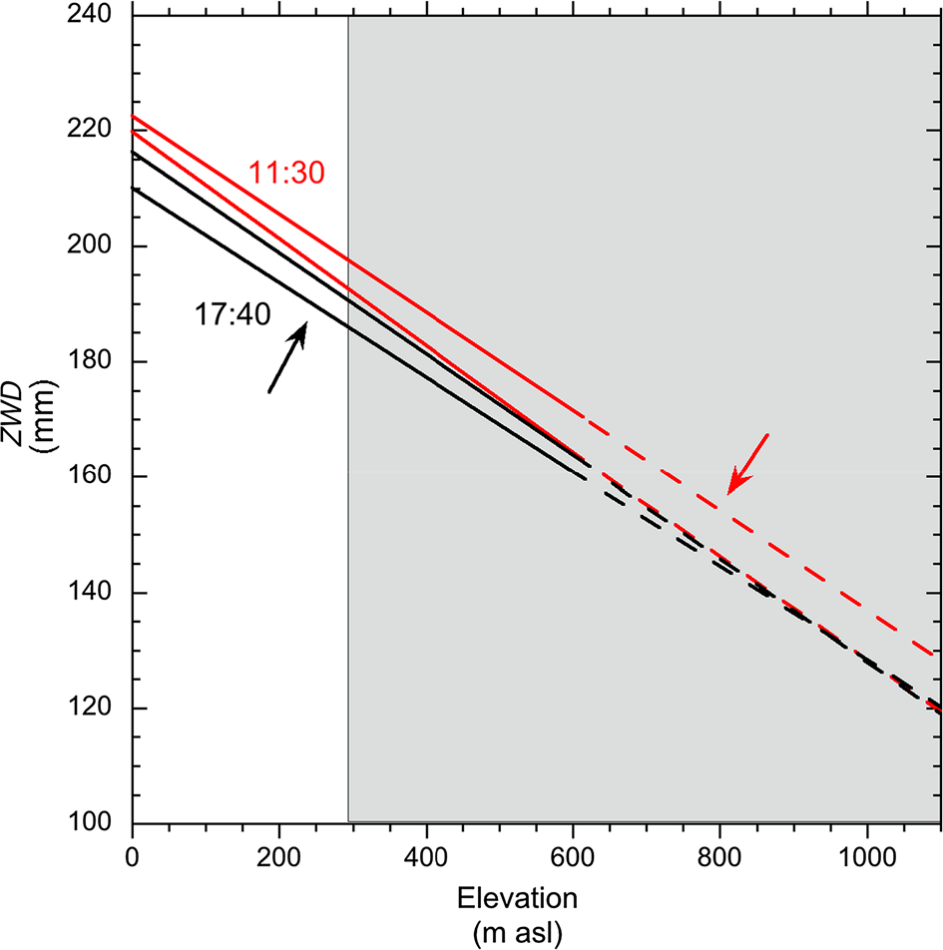
*ZWD* values plotted against receiver elevations calculated from the 14-receiver GPS network on Montserrat for two periods. The two *red lines* bound the set of linear fits of the *ZWD* lapse rate calculated every 5 min for a 1-h period centred on 1130 LT. The two *black lines* are the equivalent for 1740 LT. The *arrows* show the senses of change of *ZWD *gradient fits over the 1-h interval. The *dashed lines* are the extrapolated curves to the top of the volcano beyond the measured elevation range 0–600 m a.s.l. of the GPS network. The *grey box* denotes the elevation range of the coherent radar surfaces

The *PWV* values from the HERM GPS site show a decreasing trend over the first part of this interval that is then reversed over the second part (Fig. 4). These GPS variable incidence angle measurements, for paths steeper than $10^{\circ }$, are used in a weighted least squares algorithm to estimate the delay in the zenith direction and derive the *ZWD* values, whilst the GPRI measurements are made from the horizontal up to elevations of $8^{\circ }$, this being the vertical angle subtended from the Montserrat Volcano Observatory site to the top of the volcano. The GPS *ZWD* values generally decrease with increasing elevation of the station. Using the range of elevations (12–587 m a.s.l.) from all 14 GPS receivers across Montserrat we can calculate an island-wide average lapse rate of *ZWD* using a linear regression fit. In the following, we extrapolate this rate from sea level to the top the volcano. We can test this *ZWD* lapse rate with a comparable measurement of specific humidity from the 1700 LT 4 August radiosonde measurement (Fig. 2); the measured decrease in specific humidity between the surface and 1500 m a.s.l. on Guadeloupe was about $11\hbox { g kg}^{-1}$ . Assuming linearity to the same altitude of the GPS *ZWD* lapse rate (Fig. 6) gives about *PWV* = 19 mm. Ruckstuhl et al. (2007) demonstrated a set of linear relationships between *PWV* and specific humidity for a range of environments. Using their data from Payerne, Switzerland at about 500 m a.s.l. and under cloudy sky conditions gives an expected *PWV* value of about 26 mm (compared to our 19 mm) for a specific humidity of $11\hbox { g kg}^{-1 }$.

We now test how the change in stratified refractivity up the slope of the volcano measured by the network of GPS receivers compares with the GPRI-measured phase change represented in the interferogram of Fig. 5. The *ZWD* lapse rates are calculated every 5-min for two 1-h periods centred on the acquisition times of the interferogram (1130 and 1740 LT). The ranges of linear fits for these two periods is shown in Fig. 6, with the fits at the exact image times sitting near the middle of both ranges. The lapse rate at 1130 LT, of $0.0883\hbox { mm m}^{-1}$, is greater than at 1740 LT, $0.0825\hbox { mm m}^{-1}$, a differential rate of $0.0058\hbox { mm m}^{-1}$, and this varied by a few tens of percent over the 1-h period. The increase in the phase delay with increasing elevation over time recorded by the GPS network is the same sense as that measured by the interferogram. Over the 400–800 m a.s.l. range of coherent signals from the ground, the differential *ZWD *rate over the 6-h interval gives a delay of 2.3 mm. The slant paths of the GPRI measurements generally increase in range (4–5.5 km) as they increase their inclination ($0^{\circ }$–$8^{\circ }$) and the elevation of the ground they intersect rises from 300 to 1080 m a.s.l.).

**Fig. 7:**
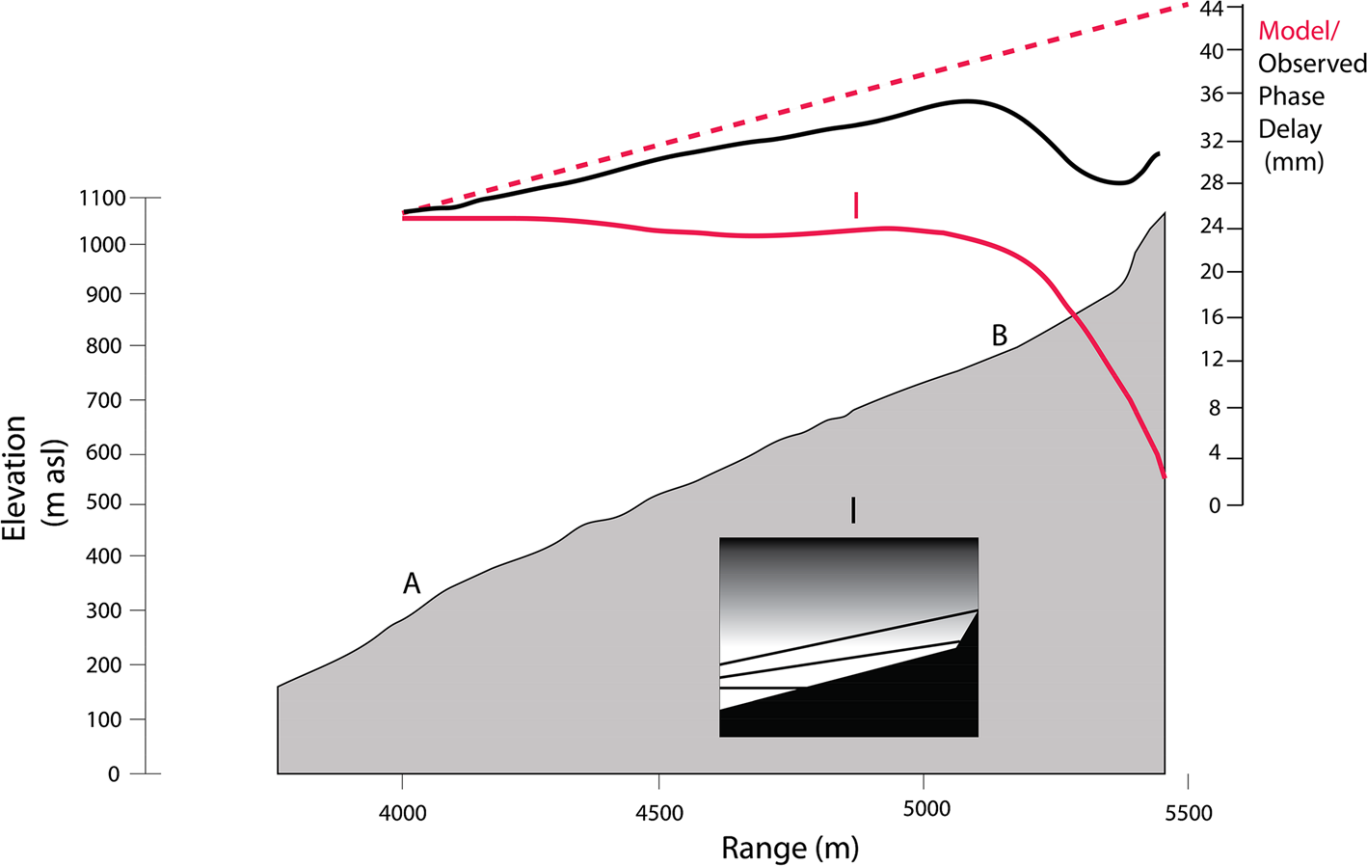
True scale cross-section of the topography (*grey area*) projected onto range through points *A* and *B*, as imaged by GPRI from the Montserrat Volcano Observatory site (Fig. 5). Elevation values are shown on the *left*-hand scale. The *black curve* is the difference in observed phase delay (mm) measured by the 6-h interferogram shown in Fig. 5. The *inset* schematic shows the modelled GPRI slant paths (*black lines*) through a stratified atmosphere of decreasing water vapour with altitude (model I). The *dashed red line* is the integrated delay for a homogeneous refractivity model with a rate of $0.00734\hbox { mm m}^{-1}$, the *solid red curve* is of the path-integrated delay from that model with an additional vertical delay rate of $0.0058\hbox { mm m}^{-1}$

The refractivity along these slant paths can be calculated using Eq. 1, if the temperature, dry-air pressure, water vapour pressure (humidity) and liquid water content along those paths are known. In relatively flat terrain and over short distances (few hundred metres), it is sometimes assumed that the atmosphere is homogeneous and that it is the range distance that determines the change in refractivity, creating a linear ramp in range (e.g. Pipia et al. 2008). However, for a vertically stratified atmosphere intersected by steep topography, as in our case, we expect the change in refractivity vertically to play a role. Iglesias et al. (2014) showed that this will generally be the case, and that the phase difference contribution from water vapour in the atmosphere, ${\varPhi }_\mathrm{ATM}$, at a given time, *t*, for a given location element, *k*, is given by,

2 $$\begin{aligned} {\varPhi }_{\mathrm{ATM}, k }(t) = 10^{-6} (4\uppi {f_c} /\hbox {c}) (N_\mathrm{R}(t) r + 0.5 N_{1}(t) r_{k} h_{k}) \end{aligned}$$

where ${f_c}$ is the radar frequency, c is the speed of light, *r* is the range distance, *h* is the height and $N_\mathrm{R}$ is the refractivity at the radar, $N_{1}=(- N_\mathrm{s}\,\upalpha )$ where $N_\mathrm{s}$ is the value of the refractivity at the volcano surface and $\upalpha$ is a height scale factor in units of $\hbox {km}^{-1}$. The first variable term in brackets in Eq. 2 represents the linear increase with range, whilst the second term represents the change with altitude. For different atmospheric conditions at different times, $t_{1}$ and $t_{2}$, the (unwrapped) differential phase of an interferogram is given by,

3 $$\begin{aligned} \Delta {\varPhi }_{ k }(t, t_{2})= & {} {\varPhi }_{\mathrm{ATM}, k }(t_{2}) - {\varPhi }_{\mathrm{ATM}, k }(t_{1})\nonumber \\= & {} \beta _{1} r_{k }+ \beta _{2} h_{k} r_{k} \end{aligned}$$

where $\beta _{1}$ and $\beta _{2}$ are defined as

4a $$\begin{aligned} \beta _{1}= & {} (4\uppi {f_c}/\hbox {c}) \times 10^{-6 } \Delta N_\mathrm{R}(t_{1,}t_{2}), \end{aligned}$$

4b $$\begin{aligned} \beta _{2}= & {} (2\uppi {f_c}/\hbox {c}) \times 10^{-6 } \Delta N_{1 }(t_{1,}t_{2}) . \end{aligned}$$

We can calculate $N_\mathrm{R}$ from Eq. 1 using the measurements recorded at the radar at the relevant times: 1130 LT ($P = 980\hbox { hPa}$, $T = 303\hbox { K}$, relative humidity = 70 %) and 1740 LT ($P = 980\hbox { hPa}$, $T = 301\hbox { K}$, relative humidity = 72 %). This yields a $\Delta N_\mathrm{R}$ value of $+7.34$, equivalent to a differential delay rate of $+0.00734\hbox { mm m}^{-1}$ at the Montserrat Volcano Observatory site. We do not have the equivalent values for the refractivity at all points on the volcano’s surface $(N_\mathrm{s})$, but we do have the vertical delay rate from the difference in the GPS *ZWD* lapse rates at these two times (Fig. 6): $+0.0058\hbox { mm m}^{-1}$. The net effect of these two rates can be calculated for any radar path, and the steeper the inclination of that path, the greater the reduction in delay for a given range.

Figure 7 shows the result of such a model (I) calculated along the A-B profile, together with the observed phase measured along the paths from A to B in the interferogram. The observed phase delay in the interferogram reaches a maximum near site B and then declines steeply. The expected behaviour for an atmosphere with a uniform refractivity delay of $0.00734\hbox { mm m}^{-1}$ and two-way travel is shown by the dashed red line in Fig. 7. The solid red curve shows the reduction of delay on higher paths as the average refractivity falls with elevation. The disparity between the observed and modelled curves may be caused by an incorrect balance of the two delay rate terms. This might be because the assumption of an island-wide linear delay lapse rate is incorrect for this part of the island. Also the upper part of the volcano may be subject to very strong lateral variability of water vapour due to dynamic factors.

This demonstrates two important points. Firstly, it is the temporal variability of the vertical gradient of water vapour content (and phase delay), not the absolute amount, that controls the kilometre-scale structure of the refractivity field. Secondly, the phase field around the lava dome (above 800 m a.s.l.) probably does not fit a linear, vertically stratified model of water vapour delay and is more complex than lower on the talus slopes.

**Fig. 8:**
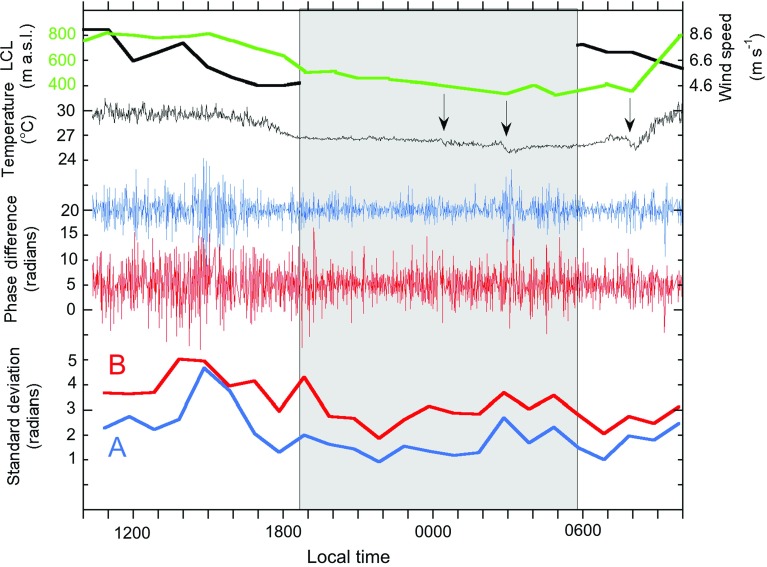
24-h time series of phase differences in radians between each 1-min interval interferogram measured at sites *A* and *B*. Below the raw data curves for *A* (*blue*) and *B* (*red*) are the standard deviation curves calculated from hourly bins. The *thin black curve* is of 1-m air temperature measured at the MVO site. Note the sudden negative deflection of this curve caused by brief rain showers (*arrows*). The *thick black curve* is the daytime wind speed recorded every hour at Gerald’s airport, 10 km north of the volcano. The *green curve* is the lifting condensation level (LCL). Night is shown by the *shaded area*

### One-minute Interferograms

**Fig. 9:**
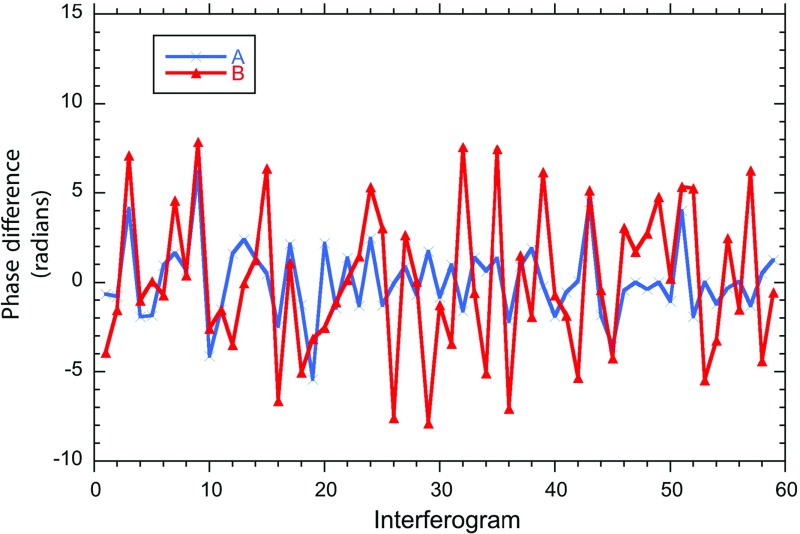
Time series of 1-min phase differences from 1620 LT to 1720 LT on 4 August from the two sites *A* and *B*. The close correlation between the two signals for the first ten interferograms breaks down and the two series are poorly correlated thereafter

Figure 8 shows the phase difference variability at 1-min intervals over the 24-h period for locations A and B. The phase variability at B is consistently greater than at A. This is because B is about 400 m higher and 1 km further from the radar than A, and is also adjacent to, and downwind from, the volcano’s gas plume. The 24-h standard deviations of phase are 2.14 radians at A and 3.34 radians at B. Standard deviations calculated over hourly intervals range from 0.92 to 4.7 radians for A and from 1.87 to 5.02 radians for B. Daytime variability at both sites is generally higher than at night with standard deviations of 1.59 and 2.89 radians for A and B at night and 2.37 and 3.59 radians for A and B in the day respectively. This must be due to more vigorous turbulence in the mixed zone of the ABL during the day. Variability is highest in the early afternoon of 4 August, when it is at the same level for both A and B for 2 h. Mid-afternoon is typically the time of maximum elevation of the mixed layer due to sea breeze intrusion up the volcano, though we do not have an independent measurement of this. For the rest of the day the phase standard deviation at B is 1–2 radians greater than at A (Fig. 8). At times the variation in phase at A and B can be seen to be correlated (e.g. in interferograms 1–10, Fig. 9), but at other times, particularly when the variability is high at B and low at A, there is a lack of correlation (Fig. 9). The reduction in wind speed between 1000 and 1800 LT (Fig. 8) may have been a factor in the reduction of phase variability between 1400 LT and 1800 LT (Iannini and Monti Guarnieri 2011).

The time series of phase changes also shows some features associated with rainfall. In particular at A (and perhaps at B) there is a clear increase in phase variability as a rain shower passed at about 0300 LT (Fig. 8) and perhaps also later at about 0800 LT. After the 0300 LT shower, the phase variability of both A and B remained higher than the previous interval for the next 3 h. The lifting condensation level curve for the Montserrat Volcano Observatory site (calculated using the Lawrence method) is asymmetrical about the nightime minimum, falling quite smoothly from 1500 LT on 4 August, but rising more much more erratically after 0300 LT on 5 August.

**Fig. 10:**
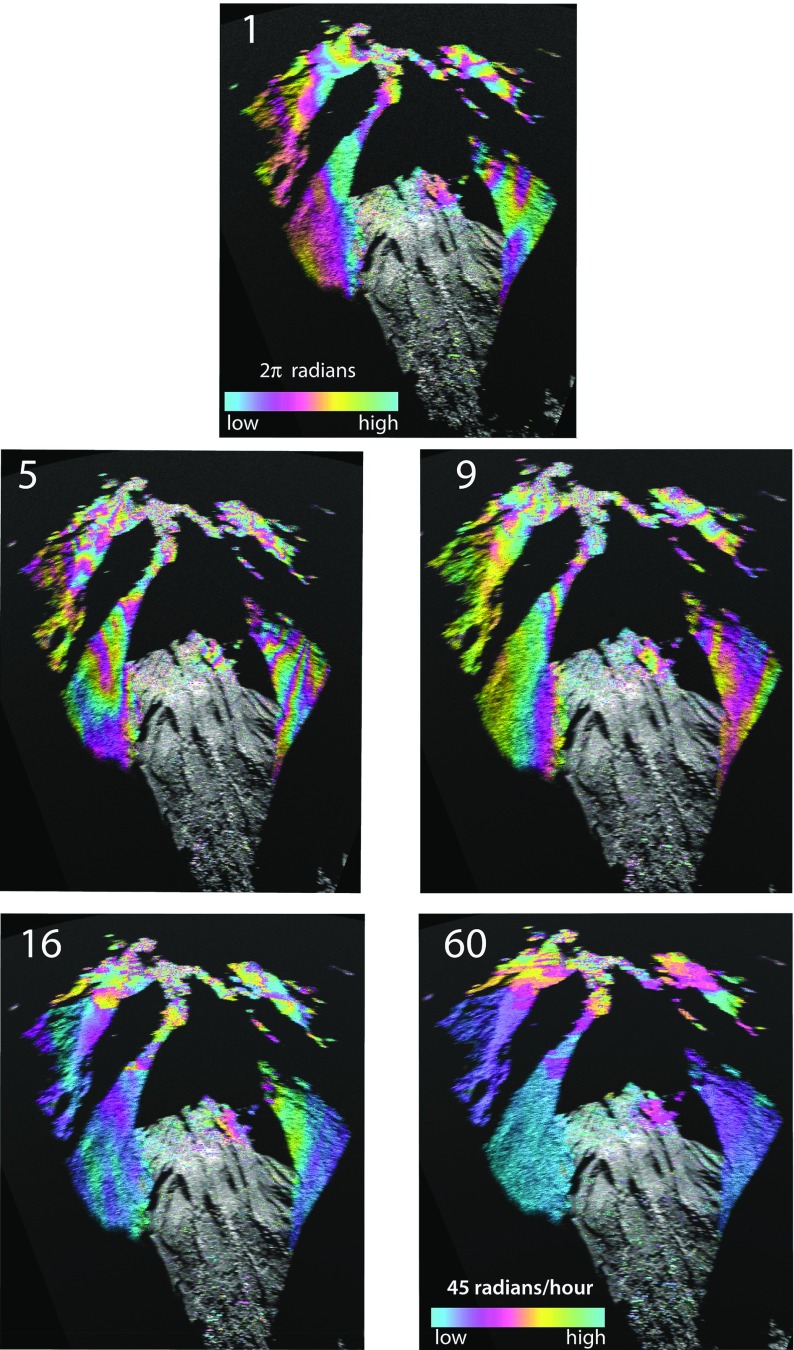
Averaged sets of 1-min interferograms of Soufrière Hills Volcano, centred on 1130 LT 4 August and taken from the Montserrat Volcano Observatory site. The number of images used in the temporal averaging is shown in *white*

### Averaged Interferograms

Averaging the phase differences in time to give a rate of phase change across a series of 1-min interferograms will tend to reduce the effect of spatially random variations of water vapour such as might arise from local thermal convection, but tend to enhance features that have greater longevity in the atmosphere. In Fig. 10 we have averaged four series of interferograms over intervals of 5, 9, 16 and 60 min, all centred on 1130 LT 4 August. The un-averaged 1130-1131 LT interferogram shows a gradient on the eastern side of the volcano. The 5-min phase rate interferogram (Fig. 10) shows a stronger, more complex, elongate pattern with an overall reversal and increase of gradient relative to the un-averaged interferogram. The coherence in the 5-min interferogram is worse than in the 1-min case on and around the dome. The phase gradients lessen and become more diffuse in the 9-min interferogram, a trend that continues in the 16-min case and into the 60-min case. In the latter, the phase gradients are gradual on the talus covered slopes. Coherence on the dome is still poor, worse than the 1-min case. During the daytime the rate of change of water vapour seems to increase over periods of a few minutes and it is only over averaging periods of several tens of minutes to an hour that smoother patterns appear.

**Fig. 11:**
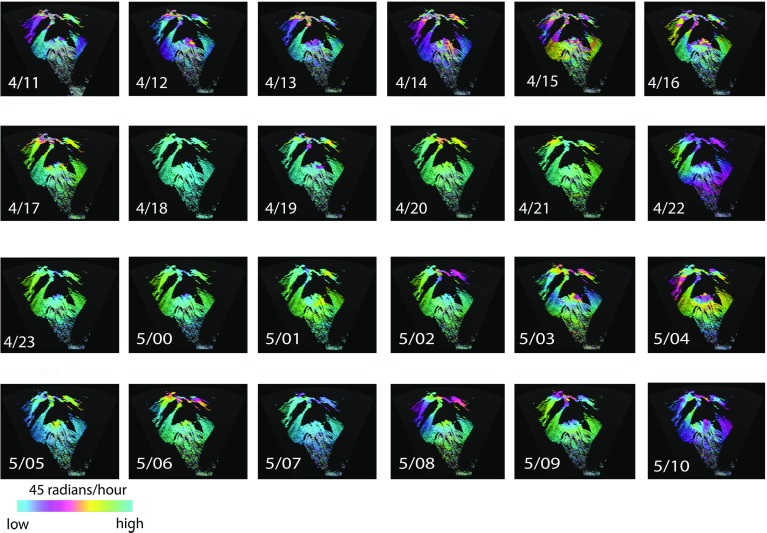
24 interferograms of Soufrière Hills Volcano taken from the Montserrat Volcano Observatory site, created by averaging 1-min interferograms in hourly (*n*) stacks [*n*:20 to ($n+1$):20] on 4–5 August 2013. Image labels give the day/($n+1$) hour in local time

**Table 2:** Phase rate and phase rate standard deviation (p.r.s.d) differences measured as hourly averages in radians $\hbox {h}^{-1}$ between site A and near the top of the dome on 4 and 5 August

Day:hour	4:12	4:13	4:14	4:15	4:16	4:17	4:18	4:19	4:20	4:21	4:22	4:23
Phase rate diff	$+5.4$	0.0	$+1.2$	$+5.8$	$-5.4$	$+4.6$	$+6.2$	$+2.9$	$+10$	$+17$	$+4.2$	$-4.2$
p.r.s.d.diff	$+16$	$+30$	$+37$	$+31$	$+21$	$+32$	$+3$	$+34$	$+20$	$+6$	$+6$	$+5$

Day:hour	5:00	5:01	5:02	5:03	5:04	5:05	5:06	5:07	5:08	5:09	5:10	
Phase rate diff	$-2.5$	$-0.8$	$-11$	$-13$	$-11$	0.0	$-4.2$	$+5.0$	$+3.7$	$-14$	$+18$	
p.r.s.d.diff	$+2$	$+6$	$+25$	$+17$	$+21$	$+27$	$+29$	$+24$	$+18$	$+24$	$+7$	

**Fig. 12:**
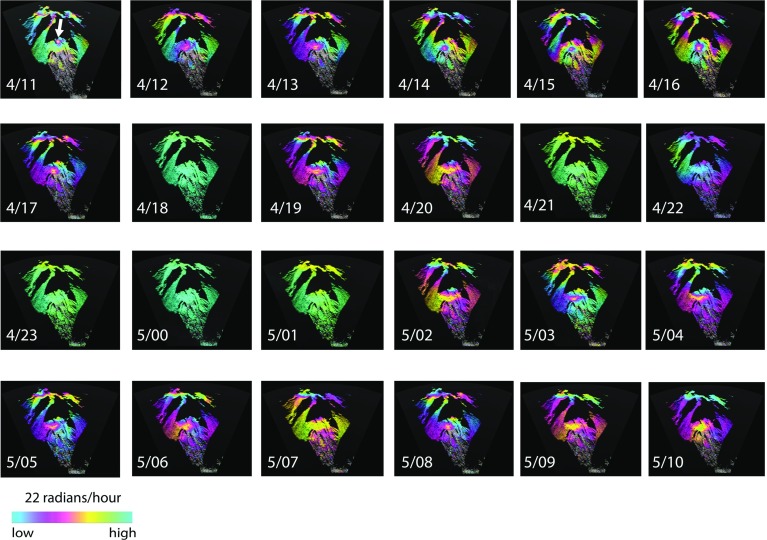
24 images of phase rate standard deviation (p.r.s.d.), derived from the interferogram stacks shown in Fig. 11 (note change of scale). The *white arrow* in 4/11 shows the position of the circular feature discussed in the text. Image labels as for Fig. 11

A full 24-h set of 1-h averaged interferograms has been created (Fig. 11). During daylight hours, the hourly-averaged interferograms tend to show considerable change in phase rate (p.r.), particularly at far range, on and around the lava dome. Poor phase unwrapping is noticeable around the dome in some of the daytime stacks, for example those of 4/15 and 4/16 (Fig. 11). There is a striking reduction in variability in the hour before sunset (at 1850 LT), on 4 August (4/18, Fig. 11) that lasts until 0200 on 5 August (5/02, Fig. 5). Generally, the daytime hours are much more variable than those in the nightime. The temporal variability of the phase from each 1-min interferogram to the next is represented in Fig. 12 by images of the phase rate standard deviation (p.r.s.d.) calculated over an hour. Such images can be interpreted as a proxy for the local strength of turbulence within the ABL. As with the phase rate images (Fig. 11), the p.r.s.d. images show an abrupt reduction in variability at sunset (4/18, Fig. 12) and an increase in variability after 0200 LT on 5 August (5/02, Fig. 12). During the daylight hours of 4 August there is a distinct, bulls-eye feature in the p.r.s.d. images, about 500 m across with lower values ($\approx$10 radians/h) of p.r.s.d. at its centre, at 4 km distance on Gages Mountain (4/11–4/17, Fig. 12). It reappears in the daylight hours of 5 August, but less distinctly. We interpret this as a quasi-permanent atmospheric feature that appears during formation of the daytime mixed layer, in the same location, suggesting control by a turbulent feature driven by orography; perhaps a vortex related to trade wind flow around Gages Mountain.

Table 2 lists the phase rate and p.r.s.d. differences measured from near the bottom of the talus (site A) to the highest point on the volcano (top of the dome) that is continuously coherent, corresponding to the hourly time series of Figs. 11 and 12. The p.r.s.d. values can be measured continuously, whilst the phase rate values often become incoherent towards the uppermost parts of the volcano (Fig. 11), particularly on the dome, and cannot be measured fully. The values of p.r.s.d. all increase with range and elevation, whilst the phase rate values are of mixed sign.

**Fig. 13:**
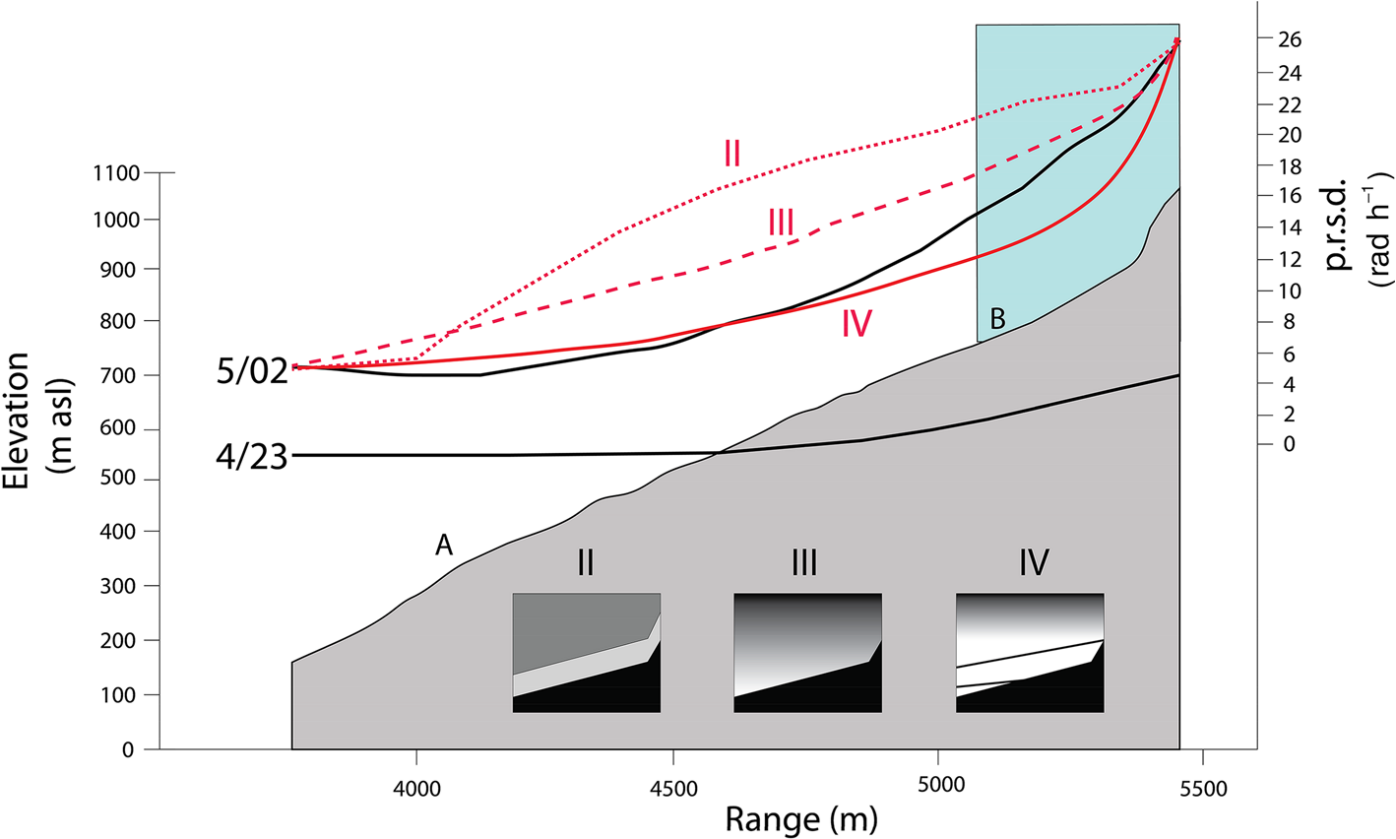
True scale cross-section of the topography (*grey*) projected onto range imaged by the GPRI from the Montserrat Volcanic Observatory site. The measured p.r.s.d. gradients along this section are shown as continuous black curves for two 1-h intervals (4/23 = 2220–2320 LT 4 August and 5/02 = 0120–0220 LT 5 August) representing low refractive variability (4/23) and high refractive variability (5/02). II, III and IV (*fine dashed*, *coarse dashed* and *continuous red curves* respectively) are the results (normalized to 5/02) from three numerical models of the ABL described in the text and illustrated graphically by the three *insets*. The two lines in *inset IV* represent the range of inclinations of radar lines of sight. The *blue area* in the main profile indicates the region adjacent to the volcanic plume

In Fig. 13 profiles in slant range from site A through site B are shown for hour 4/23 (low variability conditions) and hour 5/02 (high variability conditions). The 5/02 conditions could be due to katabatic flow or synoptic-scale variations. Both these hours show that phase variability does not increase linearly with slant range, which we might expect if we were detecting more elements of turbulence randomly distributed throughout the ABL. They do show that variability increases at increasing rates as the top of the volcano is approached with increasing line-of-sight inclinations.

We have tested three numerical models (II, III, IV) of the structure of the ABL against these observations of p.r.s.d. Lines-of-sight from the Montserrat Volcano Observatory to the base of the talus at a range of about 4000 m and to the top of the dome at 5450 m increase by $8^{\circ }$ (Fig. 13, inset IV), and the steepest lines-of-sight encounter p.r.s.d. values greater by a factor of 5 than those on the near-horizontal paths. Model II, has a two-component model with a 200 m thick uniform surface layer below a uniform mixed layer with a p.r.s.d. ratio of 1:2 between the two. Model III is a single mixed layer whose refractivity variability increases linearly with altitude and Model IV is also a single mixed layer, but with variability increasing upwards logarithmically (Fig. 13). Model II, normalized to the observed range of 5/02, is a poor fit to the observations. Models III and IV are better fits to 5/02, suggesting a positive gradient of refractivity variability with altitude. An alternative explanation that we cannot explore here is that variable local flow patterns around the complex cliff topography of the lava dome may be responsible for some increased turbulence-induced delays, seen for example in the hourly averages of p.r.s.d. of the dome region in Fig. 12.

### The Volcanic Plume

The volcanic plume consists of hot gases, mainly water vapour. Both the increase in temperature of the atmosphere as the hot gases mix with it and the addition of water vapour will tend to increase the variability of atmospheric refractivity (e.g. Houlié et al. 2005). The trade winds carried the volcanic plume from east to west during our measurements, as shown in Fig. 3. The sources of the plume are mainly scattered fumaroles with temperatures up to about $600\,^{\circ }\hbox {C}$, in the upper part of the 2010 crater, on the remnants of the partially collapsed lava dome. The visible, condensed part of the plume commonly rises buoyantly by $< 500\hbox { m}$ above the crater rim before being advected to the west. Thus the plume usually occupied part of the radar lines-of-sight from the Montserrat Volcano Observatory site to the dome, site B, Chances and Spring Ridge (Fig. 3). If the plume were constant in flux and location then it would have no effect on the interferograms. During lava extrusion the measured gas flux was variable over periods of tens of minutes (Young et al. 2003) and was almost certainly not constant during our measurements. The averaged $\hbox {SO}_{2}$ flux in the plume measured by the Montserrat Volcano Observatory over eight daytime hours on 4 August 2013 was about 250 tonnes/day. The corresponding water component of the plume emitting into the atmosphere is not known directly and is assumed here to be in the form of water vapour, but is probably about 500 times that of the $\hbox {SO}_{2}$ flux: 125,000 tonnes day$^{-1}$ ($\approx$1450 kg s$^{-1}$). If we assume a plume ($\hbox {air density} = 1.225\hbox { kg m}^{-3}$) of diameter 0.5 km moving at $10\hbox { m s}^{-1}$, this would have a flux of $\approx$2.4 $\times 10^{6}\hbox { kg s}^{-1}$. Adding $1450\hbox { kg s}^{-1}$ of volcanic water vapour to this plume would only raise the humidity mixing ratio by $0.6\hbox { g kg}^{-1}$ from an ambient value in the range 6–$12\hbox { g kg}^{-1}$. However, local perturbations of flux, particularly close to the fumarole sources could conceivably raise this value by an order of magnitude and produce a general increase in refractivity in the plume and increased refractivity variations detectable by GPRI.

During vigorous explosive eruption of lava and ash the plume temperature increases (by several tens $^{\circ }\hbox {C}$) and can become the dominant factor in raising the refractivity (Houlié et al. 2005). But for Soufrière Hills Volcano in 2013, any elevation in plume temperature must be of a few $^{\circ }\hbox {C}$ at most, at heights of hundreds of metres above the ground.

Site B on the 2010 crater rim is prone to significant plume water vapour variability, as well as that due to meteorological water vapour. It is possible that the large amplitude swings in phase difference (e.g. Fig. 9), uncorrelated with those affecting site A lower down and out of reach of the plume, are due largely to the variable water vapour flux within the plume. Beyond site B the radar propagation paths extends another 400 m or so to the top of the lava dome. These paths could also be prone to modulation by the plume’s variable passage. However, perhaps we might expect to see evidence of the plume’s continuing variability, independent of the stable layer during the night. But the hourly interferograms, showing very low phase rate variability on high elevation paths at 4/18, 4/23 and 5/00 for example (Fig. 12), suggest not.

## Summary and Fit to the ABL

Let us first consider the known limitations of the study. We have not sampled the upper half of the ABL at Montserrat, though the radiosonde-detected inversions are in rough agreement with an ABL 2 km in height. We have made no attempt to account for the effects of liquid water on the refractivity (Eq. 1), as it is of much smaller magnitude than water vapour in most circumstances (Hanssen 2001). We note that during the arrival of more humid air on 5 August, the 1-min interferograms showed more variation after brief rain showers. This could be due to liquid water effects or higher water vapour, perhaps associated with latent heating during change in the phases of water. The GPRI cannot capture the structure of the water vapour field above the dome or other parts above the skyline. The complexities of the water vapour field around and just below the dome detected by GPRI must be part of a fuller flow field that extends around and above it. Finally, whilst we might expect orographic winds on the volcano to affect the refractivity, perhaps in the valleys, we have no direct evidence of them.

Of the three types of forcing of the water vapour field that were anticipated: diurnal radiative forcing, synoptic-scale atmospheric change and volcanic plume variation, the effects of the first two were easier to address, the plume provides more ambiguous evidence. Two patterns dominated the GPRI measurements: the variability of refractivity is generally much less at night than during the day, and refractive variability increases with distance (slant range). These patterns have been observed before, but we have also made some telling new observations.

Whilst the water vapour variability was generally reduced at night, there were periods of higher values. This was particularly so after 0200 LT on 5 August when short periods of higher humidity (with short rain showers) began to traverse Montserrat as part of a synoptic weather system. The rain shower at 0300 LT was accompanied by a distinct pulse in the water vapour variability (Fig. 8). Two of the periods of lowest water vapour variability occurred in the hour before sunset and the hour after sunrise. The reason for this is unclear.

Independent GPS measurements showed that the water vapour content of the atmosphere rose by about 40 % over the observation period during the passage of the synoptic-scale feature (Fig. 4). This is not reflected by an equivalent general rise in the phase variability measured by GPRI. This suggests that either the water vapour increase was entirely borne by the upper part of the troposphere not sampled by GPRI, or more likely, that water vapour variability is controlled mainly by the diurnal (and plume) processes. The phase variability was greatest on the afternoon of 4 August, with declining winds speeds. This suggests that reducing turbulent kinetic energy, driven by lower wind speeds, was partly responsible (Barucci et al. 2010).

A roughly circular feature of reduced phase variability recurs to the north of Gages Mountain and is strongest in the daytime data of 4 August. It was also observed at other times outside this campaign and its location is well away from the volcanic plume. We interpret it as a local airflow feature caused by interaction of the trade wind and the topography of Gages Mountain. High resolution 3D modelling could be used to test the conditions of its formation.

During this observation period the volcanic plume maintained a fairly constant trajectory and thus we were not able to measure the phase magnitude of a plume/no-plume change in the radar paths. Extrapolations from observed rates of sulphur dioxide emission rates in 2013 suggest anyway that the plume might only be able to contribute a few percent to the humidity mixing ratio of the ambient air, perhaps greater at lower levels, closer to the fumaroles. Whilst p.r.s.d. may increase on paths through the plume (e.g. at site B), the minute-to-minute correlations with site A, out of the plume that are sometimes seen (Fig. 8) suggests both non-plume and plume sources of water vapour variability.

Several studies of ground-based radar interferometry (e.g. Iannini and Monti Guarnieri 2011) have observed increased phase differences with slant range and then used models with a linear dependency on range. In contrast, we see evidence from modelling of a 6-h interferogram that the radar path inclination through a stratified atmosphere plays a major role in determining the structure of the refractivity field, as advocated by Iglesias et al. (2014). That is, the more the radar path traverses higher levels of the mixed zone in the day and the residual zone at night, the greater the p.r.s.d. values measured. Models in which the water vapour variability increases with altitude give better fits to the p.r.s.d. measured in profile up the volcano than a neutral or two-layer model (Fig. 13).

**Fig. 14:**
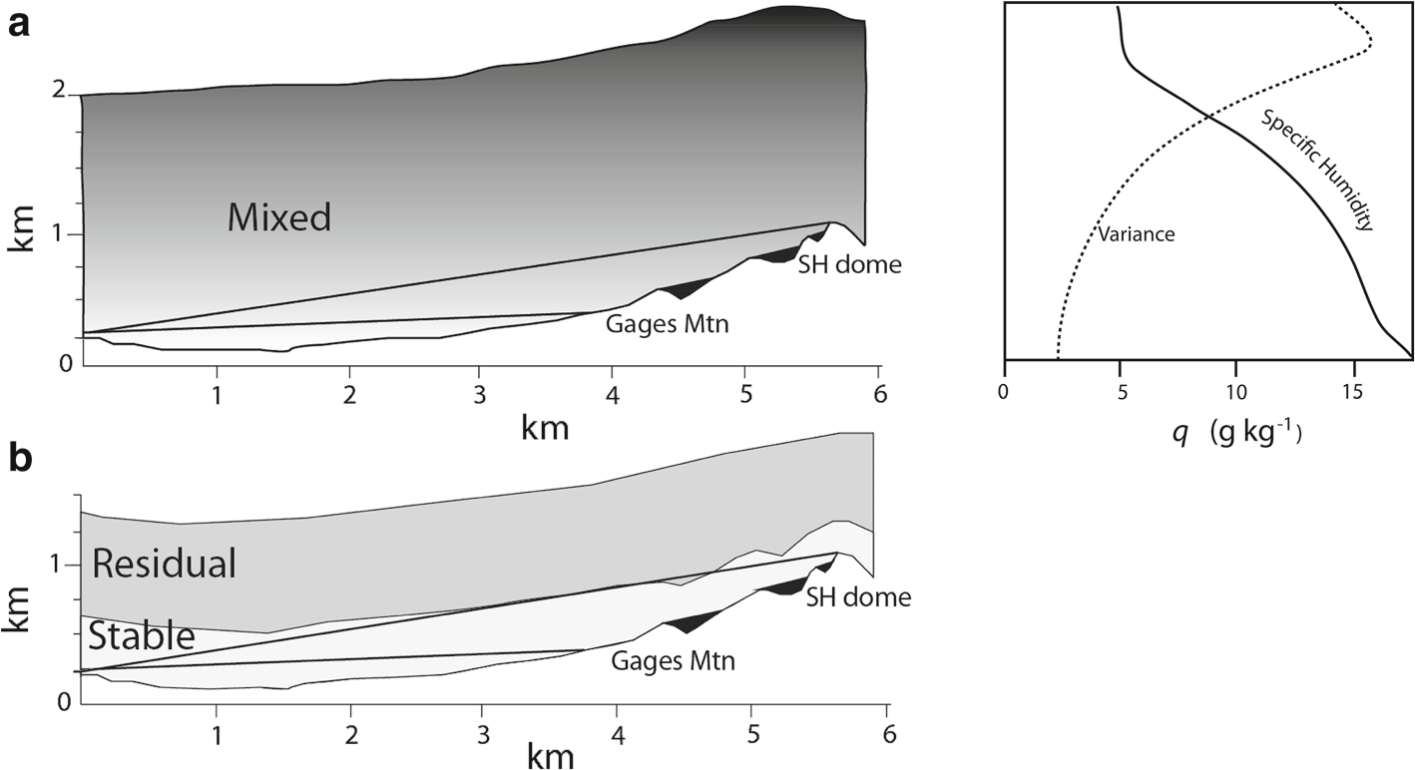
Schematic topography-atmosphere cross-sections from the Montserrat Volcanic Observatory observing site (*left* side of figure) to the summit of the volcano 5.5 km to the south-south-east are shown under idealised ABL conditions for day (**a**) and night (**b**). The straight lines are radar lines-of-sight discussed in the text and the areas of *black* are those occluded from radar illumination. To the *upper right* is a profile of the specific humidity through the daytime ABL based on the BOMEX results (Siebesma et al. 2003), which can be regarded as the baseline ocean ABL state for the atmosphere around Montserrat. The variance of the specific humidity is shown schematically (no units) based on observations and modelling (Stull 1988; Wulfmeyer et al. 2010). This shows very high levels of specific humidity variance around the entrainment layer falling to much lower levels at the base of the mixed layer

In Fig. 14 we show schematically how the diurnal evolution of the ABL could explain the GPRI-observed phase variability. During the day, the radar paths to lower elevations around Gages Mountain are mainly within the lowermost part of the turbulently mixed layer (Fig. 14a), whilst the paths to the top of the volcano are in the higher, more variable, part of the mixed layer. Increasing water vapour variance upwards in this layer produces the observed patterns of refractive change during the day. Observations (Wulfmeyer et al. 2010) and modelling (Stull 1988) elsewhere indicate that upward-increasing variance of specific vapour humidity in the mixed layer is to be expected, which is consistent with our observations. Paths to the ground surface above 800 m a.s.l. and 5 km range also pass through the volcano’s plume and this may contribute a small component to this pattern. During the night the lower paths are within the stable layer that has a low variance in water vapour and the upper paths are only partly within the residual layer (Fig. 14b). We have not tried to incorporate orographic wind data in these models because we have no supporting data. Similarly we would need other lines of evidence to properly parameterise the degree of turbulence of the ABL layers. Our wider GPRI and GPS dataset, together with high resolution modelling of the atmosphere, should help us to refine these ideas.

Given the variability of the refractivity revealed here, how useful would the GPRI data be for measuring ground motions due to volcanic activity on Soufrière Hills Volcano (Odbert et al. 2014)? The island-wide monitoring of deformation is undertaken by the Montserrat Volcano Observatory using GPS, and GPRI does not help in that regard. Where it could help is in measuring near-field deformation (with magnitudes of a few tens of mm) close to the volcanic vent (<2 km radius), where it is dangerous to work on the ground. Obviously, the nocturnal interludes with low atmospheric refractive variability would be the target times for such measurements. We have shown, in one example, how hour-long averages of data are likely to yield superior signal-to-noise results compared to averages of a few minutes. Hour-long intervals with p.r.s.d. values of 1–5 radians that were attained several times on 4/5 August 2013 are equivalent to about 1.5–7 mm of ground motion. This is much smaller than the expected magnitude ($\approx$20 mm) of near-field ground differential motion distributed over several hours.

## Conclusions

Our measurements of refractivity change over the Soufrière Hills Volcano for a period of one day showed that water vapour variation was considerable. We conclude that the concept of a structured ABL is a valuable means of exploring those refractivity changes. We found that:

1. A ground-based interferometer with a 1-min interval imaging capability enabled us to sample and characterise the refractivity changes due to variable water vapour in the atmosphere over the volcano.
2. In our case, three forcing agents were responsible for most of the variations in water vapour: a tropical diurnal cycle, the passage of a synoptic-scale weather system and the vagaries of the volcanic plume.
3. A rise in atmospheric humidity by 40 % over several hours had little obvious effect on phase gradients or temporal variability.
4. The standard deviation of measured radar phase due to water vapour variability in 1-min interval interferograms over periods of a 1-h was in the range 1–5 radians.
5. Variations of refractivity were generally lowest at night and greatest in mid-afternoon. The nocturnal record was interspersed with periods of higher variability, some of which can be related to the passage of a synoptic-scale weather system.
6. Phase gradients and temporal variability increased with slant range and elevation. Models of these indicate a strong vertical gradient in refractivity perturbations.
7. Estimates of the phase delay due to the changing volcanic plume suggest a minor role.
8. The diurnal cycle of the ABL with a daytime turbulent mixed layer and nightime residual/stable layers fit the observations well and could form the basis of a more quantitative model of the observed pattern of refractive changes over the course of a day.
